# Multiple Genetic Interaction Experiments Provide Complementary Information Useful for Gene Function Prediction

**DOI:** 10.1371/journal.pcbi.1002559

**Published:** 2012-06-21

**Authors:** Magali Michaut, Gary D. Bader

**Affiliations:** 1 The Donnelly Centre, University of Toronto, Toronto, Ontario, Canada; 2 Department of Molecular Genetics, University of Toronto, Toronto, Ontario, Canada; 3 Department of Computer Science, University of Toronto, Toronto, Ontario, Canada; University of Zurich and Swiss Institute of Bioinformatics, Switzerland

## Abstract

Genetic interactions help map biological processes and their functional relationships. A genetic interaction is defined as a deviation from the expected phenotype when combining multiple genetic mutations. In *Saccharomyces cerevisiae*, most genetic interactions are measured under a single phenotype - growth rate in standard laboratory conditions. Recently genetic interactions have been collected under different phenotypic readouts and experimental conditions. How different are these networks and what can we learn from their differences? We conducted a systematic analysis of quantitative genetic interaction networks in yeast performed under different experimental conditions. We find that networks obtained using different phenotypic readouts, in different conditions and from different laboratories overlap less than expected and provide significant unique information. To exploit this information, we develop a novel method to combine individual genetic interaction data sets and show that the resulting network improves gene function prediction performance, demonstrating that individual networks provide complementary information. Our results support the notion that using diverse phenotypic readouts and experimental conditions will substantially increase the amount of gene function information produced by genetic interaction screens.

## Author Summary

Genetic interactions map functional dependencies between genes, under a given phenotype. In the budding yeast *Saccharomyces cerevisiae*, most genetic interactions have been measured under a single phenotype - growth rate in standard laboratory conditions. Recently, genetic interactions have been collected under different phenotypic readouts and experimental conditions. How different are these networks and what can we learn from their differences? We analyzed quantitative genetic interaction networks mapped in yeast under different experimental conditions and phenotypic readouts and found that they provide significant unique information. We next asked if this unique information is complementary. As a measure of complementarity, we asked if combining networks mapped under different experimental conditions could improve gene function prediction. Two genes that genetically interact with a similar set of genes (two genes with similar genetic interaction profiles) are more likely to be in the same pathway or complex and this can be used for gene function prediction. We found that combining multiple genetic interaction profile correlation networks using a simple ‘maximum correlation’ approach improved gene function prediction, demonstrating that the networks provide complementary information. Thus, using diverse phenotypic readouts and experimental conditions will likely increase the amount of information produced by genetic interaction screens.

## Introduction

A genetic interaction is defined as an unexpected phenotype for a combination of mutations given each mutation's individual effect [1]. Genetic interactions provide valuable information about gene function and are useful to study the organization of biological processes in the cell [2]. Experimental techniques are now available to map genetic interactions at a large scale, in particular in *Saccharomyces cerevisiae* [3]. A genetic interaction is obtained in an experiment using a particular phenotypic readout and set of experimental conditions in a given species. Typically, a single, easy to observe phenotype, such as cell growth, is used to measure genetic interactions on a large scale [3]. As most yeast genes have no deletion mutant defect in rich media, but have a defect in at least one environmental condition [4], and individual genetic interactions change under different phenotypic readouts [5], it has been postulated that many unknown genetic interactions could be uncovered by performing the same interaction mapping experiment under different conditions [6]. However, no large-scale quantification of this effect has been undertaken. Here we ask how much more genetic interaction and gene function information is gained by mapping genetic interactions using different phenotypic readouts and experimental conditions.

A handful of recent studies have examined parts of this question. Linden et al. developed a normalization method to maximize the similarity between genetic interaction networks mapped by different laboratories so they can be combined [7], but this was only applied to networks obtained using the same phenotypic readout (growth phenotype). St. Onge et al. showed that mapping genetic interactions in multiple environmental conditions (standard laboratory and compound-induced DNA damage) provides useful information to infer functional relationships and order pathways [8], however this study was based on only 26 genes. An identical comparison involving almost 400 genes revealed differences between conditions and many (60–80%) condition-specific interactions [9], and methods have been developed to identify genetic interactions changing between conditions [10]. In a complementary approach, Carter et al. defined multiple types of genetic interactions in order to extract as much biological information as possible from raw data [11]. These studies show that changing environmental conditions and interaction definition provides additional information about genetic interaction. However, none have yet considered other aspects of experimental conditions, such as different phenotypic readouts, or how much overlap between networks is expected given known false positive and negative rates.

While most genetic interaction studies in budding yeast assess cell fitness by measuring cell growth in standard laboratory conditions, an increasing number have mapped genetic interactions under other experimental conditions. These include environmental conditions such as DNA damage [8]–[10] or low-ammonium agar [12], and phenotypic readouts such as gene expression [13], filamentous growth [12], endocytosis [5] and unfolded protein response [14] instead of normal growth. Earlier studies focused on small gene sets (less than 150) but recent studies have increased that number [5], [9], [14] to about 300–500 genes per study, which enables a systematic comparison.

**Figure 1:**
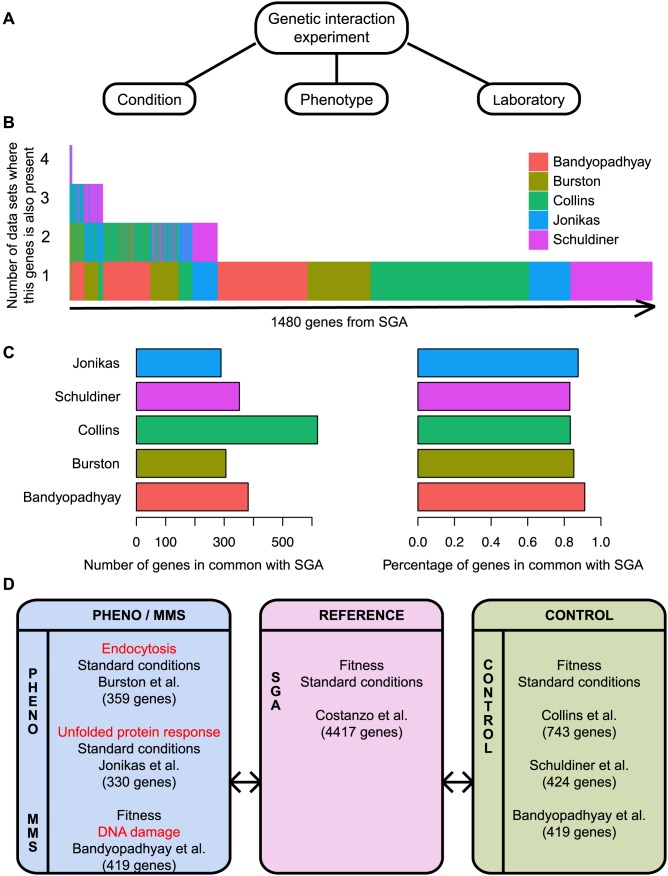
Overview of the comparison approach. A) Genetic interaction experiments differ in the phenotypic readout used, the environmental conditions and the laboratory where the experiment was conducted. B) Every network is compared to a common reference, the SGA network [3]. For each of the 1480 genes in SGA that are also present in at least another network, we show which data set considered that gene in their study. The two networks obtained in Bandyopadhyay et al. (untreated and MMS) are based on the same genes. C) The bar plots indicate how many genes are in common with the reference for each network considered. D) We compared genetic interactions mapped using different phenotypic readouts [5], [14] and in different environmental conditions [9] to the reference [3]. A set of networks mapped using similar experimental conditions was used as a control [9], [15], [16]. We also compared gene pairs tested in the reference, a control network and a network based on different phenotype or environmental condition (not shown).

We use this recently available data to conduct a systematic analysis of quantitative genetic interaction networks in budding yeast mapped under different conditions, phenotypic readouts and laboratories (Figure 1A), while considering false positive and false negative rates. We chose the largest available network as the reference [3] and compare it to a network mapped in a different environmental condition (DNA damage) [9], as well as two networks mapped using different phenotypic readouts (endocytosis and unfolded protein response) [5], [14]. A set of networks mapped under similar experimental conditions was used as a control [9], [15], [16]. We find that networks obtained in different experimental conditions overlap less than expected by chance and provide unique and complementary information. We also find that the laboratory where the experiments are carried out has an important effect on the resulting genetic interaction network. Finally, we develop a method to combine all networks together in a way that improves gene function prediction.

## Results

### Genetic interaction networks mapped under different conditions are compared to a reference network and to each other

We collected seven different quantitative genetic interaction data sets (Figure 1). Unfortunately, even though these data sets are reasonably large (more than 300 genes each, Text S1), no gene was included in all of them and only a few genes were present in four studies (Figure 1B), eliminating the possibility of a direct global comparison. However, the very large Synthetic Genetic Array (SGA) genetic interaction data set [3], which was obtained in standard laboratory conditions using colony growth as the phenotypic readout, is comprehensive enough to contain most (80–90%) of the genes tested in each of the other data sets (Figure 1C) and has a relatively high precision (0.63 for negative interactions and 0.59 for positive interactions). Thus, we used SGA as a reference and compared each of the other data sets to it (Figure 1D). This approach enables us to consider most of the genes tested in each study, though it doesn't consider possible bias from function-based gene selection across most studies. Thus, we additionally analyzed pairs of genes tested across three studies that used different phenotypic readouts and conditions.

We hypothesized that networks obtained using different phenotypic readouts or in different conditions would be more different than expected, whereas networks obtained in similar experimental conditions would be similar. To investigate the effect of using different phenotypic readouts on the resulting genetic interaction network, we compared two networks (PHENO) that used non-growth phenotypes to define genetic interactions (endocytosis defect [5] and the unfolded protein response [14]) to SGA. Both networks are independently biologically informative as shown in the original analysis [3], [5], [14]. Genetic interactions are also known to be dependent on environmental condition, such as temperature, starvation, or DNA damage induced by a small molecule [8], [9]. To investigate the effect of condition on the resulting genetic interaction network, we compared our reference SGA network, mapped in standard laboratory conditions, to the Bandyopadhyay et al. genetic interaction network, mapped in the presence of methyl methanesulfonate (MMS), a DNA damage-inducing compound [9]. The three networks obtained using different phenotypes or in different environmental conditions are referred to as the PHENO/MMS set. We also collected a set of three networks similar to the reference (similar ‘growth’ phenotypic readout and environmental conditions) obtained by other research groups, referred to as CONTROL. To perform meaningful comparisons (network of interest vs. SGA and SGA vs. CONTROL vs. PHENO/MMS), analyses were limited to the set of gene pairs tested in two or three data sets, respectively (Text S1).

### PHENO/MMS networks overlap less with the reference than CONTROL networks

In quantitative genetic interaction networks, nodes represent genes and weighted edges quantify the deviation of the double mutant phenotype from what is expected from the single mutant phenotypes. Edge weight is positive if the phenotypic readout is significantly higher than expected and negative if it is significantly lower. We treated the networks as undirected and did not consider the query or array role. We used four measures to compare networks:

1. Correlation: Spearman correlation of quantitative interaction scores, where a high value indicates two networks with highly similar quantitative genetic interactions.
2. Overlap: Amount of qualitative interaction overlap (measured using Jaccard similarity), where interactions (positive or negative) are binarized with ‘interaction’ = one and ‘no interaction’ = zero. A high score indicates that two networks generally agree on whether a given gene pair interacts or not.
3. Unique: Number of unique interactions in each network. A high number signifies large disagreement between networks.
4. Disagree: Number of interactions that disagree on interaction sign (positive vs. negative).

These measures were computed only for genes and gene pairs present in both network of interest vs. SGA and in three networks SGA vs. CONTROL vs. PHENO/MMS. We also evaluated how different the resulting measures are for a given network pair from what is expected based on a statistical model that considers known experimental interaction detection error rates.

**Figure 2:**
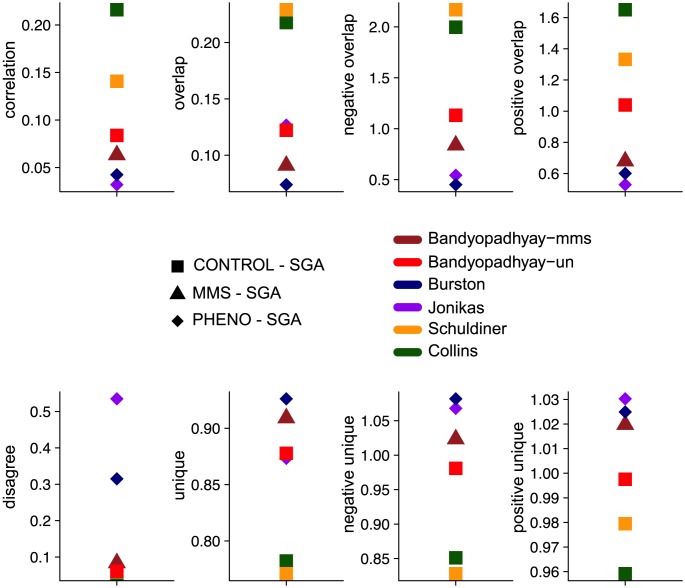
Comparison of the networks with various measures. Each square represents the comparison of a network to the reference and is colored according to the group of the networks (CONTROL, PHENO, MMS). The comparison measures are: ‘correlation’ is Spearman's correlation coefficient; ‘overlap’ is the percentage of interactions in common among all observed interactions; ‘negative (resp. positive) overlap’ is the ratio of expected/observed overlap based on our statistical model for negative (resp. positive) networks; ‘unique’ is the percentage of interactions observed in only one network among all observed interactions; ‘negative (resp. positive) unique is the ratio of expected/observed unique ratio based on our statistical model for negative (resp. positive) networks; ‘disagree’ is the percentage of interactions of different type (positive, negative) among all interactions observed in common.

Analyzing networks obtained using different phenotypic readouts, we find that SGA and PHENO networks have quantitative genetic interaction scores that are less correlated (0.037 on average) than SGA and CONTROL networks (0.13 on average) (Figure 2). This shows that SGA and PHENO networks contain different information. The lack of SGA-PHENO correlation could in part be due to error and noise differences between experiments, though the higher SGA-CONTROL correlation between networks from different research groups suggests that this is not simply due to laboratory specific effects.

We also find that SGA and PHENO networks overlap less (0.10 on average) than SGA and CONTROL networks (0.19 on average) (Figure 2). These results could be due to experimental errors in both data sets or to genuinely complementary biological information. To distinguish between these two cases, we estimated the expected level of overlap given the experimental error rates of the networks, following previous work on network error modeling [17]. Positive and negative interaction networks have different properties and error rates [3], thus we analyzed them separately. Since we limited our study to genetic interactions involving gene pairs that were tested in both data sets, the absence of an interaction indicates that no genetic interaction was detected between the corresponding two genes. This provides us with an accurate number of negatives for the error model. Based on an estimation of the error rates of the data sets, we computed the overlap expected by chance (Methods). We find that SGA and PHENO overlap less than expected (ratio observed/expected 0.53 on average, Text S1). As a control, we compare SGA to each of our ‘similar phenotype’ CONTROL networks and find that they overlap more than expected (ratio 1.55 on average, Text S1). In agreement with this, SGA and PHENO have more unique interactions and are more unique than expected while SGA and ‘similar’ CONTROL networks are less unique than expected (Figure 2, Text S1). We also found that SGA and PHENO networks disagree more on interaction sign than ‘similar phenotype’ networks (SGA vs. CONTROL) (Figure 2). Values obtained for PHENO networks are also significantly different to those of the CONTROL networks in general (Figure 2, Text S1). Taken together, we observe substantial differences between genetic interaction networks mapped using different phenotypic readouts and these are not simply due to network error rates.

We repeated the analysis on networks obtained in different environmental conditions, and found similar results: SGA and MMS have a lower correlation, lower overlap, higher unique ratio and higher disagreement ratio than networks in the control set (Figure 2). In addition, SGA and MMS overlap less and provide more unique information than expected (Text S1). Values obtained for the MMS network are also significantly different to those of the CONTROL networks in general (Figure 2, Text S1).

While we observe a consistent trend across PHENO and MMS vs. reference and CONTROL vs. reference comparisons, it is possible that function-based gene selection in PHENO, MMS and CONTROL networks could bias the data in a way that artificially causes the results we observe. To gain more confidence in our results, we additionally analyzed all gene pairs that were tested in the reference SGA network and one of the PHENO/MMS networks and one of the CONTROL networks. For the 48,499 gene pairs tested in these three categories (SGA, PHENO/MMS, CONTROL), we found that the correlation between SGA reference and PHENO/MMS is lower than between SGA and CONTROL values (paired T-test p<0.003, Figure S1). Similarly, the overlap is lower (paired T-test p<0.029) and the agree ratio is lower (paired T-test p<0.011). Each network seems to provide a similar level of unique information in this analysis, as the unique ratios are not significantly different.

Altogether, our results show that genetic interaction networks mapped using different phenotypic readouts and in different environmental conditions provide unique information.

### Networks obtained in different experimental conditions provide complementary information

We have shown that genetic interaction networks obtained under different experimental conditions (phenotype readout or environmental condition) provide unique information. We next examined if this unique information is complementary. Since a major goal of mapping genetic interactions is to discover new gene function information, we used gene function prediction performance as a measure of biological information contained in a genetic interaction network. Two genes that genetically interact with a similar set of genes (two genes with similar genetic interaction profiles) are more likely to be in the same pathway or complex [16], [18]. Thus, the function of a gene in a genetic interaction network can be predicted based on genes with similar genetic interaction profiles (a guilt-by-association approach). The quantitative genetic interaction network can be transformed into a genetic profile correlation network useful for gene function prediction by computing a correlation coefficient of the genetic interaction profiles for all gene pairs. We can then measure gene function prediction performance by holding out a fraction of a set of genes known to have the same function (e.g. cell budding), using the remaining genes to predict additional genes with the same function (based on genetic interaction profile similarity), and then assessing how many known (held out) genes were in the prediction list. This can be repeated with all available gene function categories and is automated using the GeneMANIA gene function prediction software system [19], [20].

We reasoned that if gene function prediction performance improves when genetic interaction networks are combined then they must contain complementary information. To combine a network of interest with the reference network, we computed a genetic interaction profile similarity network for each one (using Spearman correlation) and then chose the maximum correlation value for a pair of genes to include in the ‘combined’ network. To make the comparison fair, we analyzed just the set of genetic interactions tested in all the networks we compared. We quantified the utility of the individual correlation networks and the combined correlation network for gene function prediction using GeneMANIA with all available Gene Ontology (GO) terms [21]. Since we used five-fold cross validation, we limited our analysis to GO terms with at least five genes. We measured gene function prediction performance using the area under the receiver-operating characteristic (ROC) curve and the area under the precision recall (PR) curve statistic for each term in the three gene ontologies (Biological Process, Molecular Function, Cellular Component).

**Figure 3:**
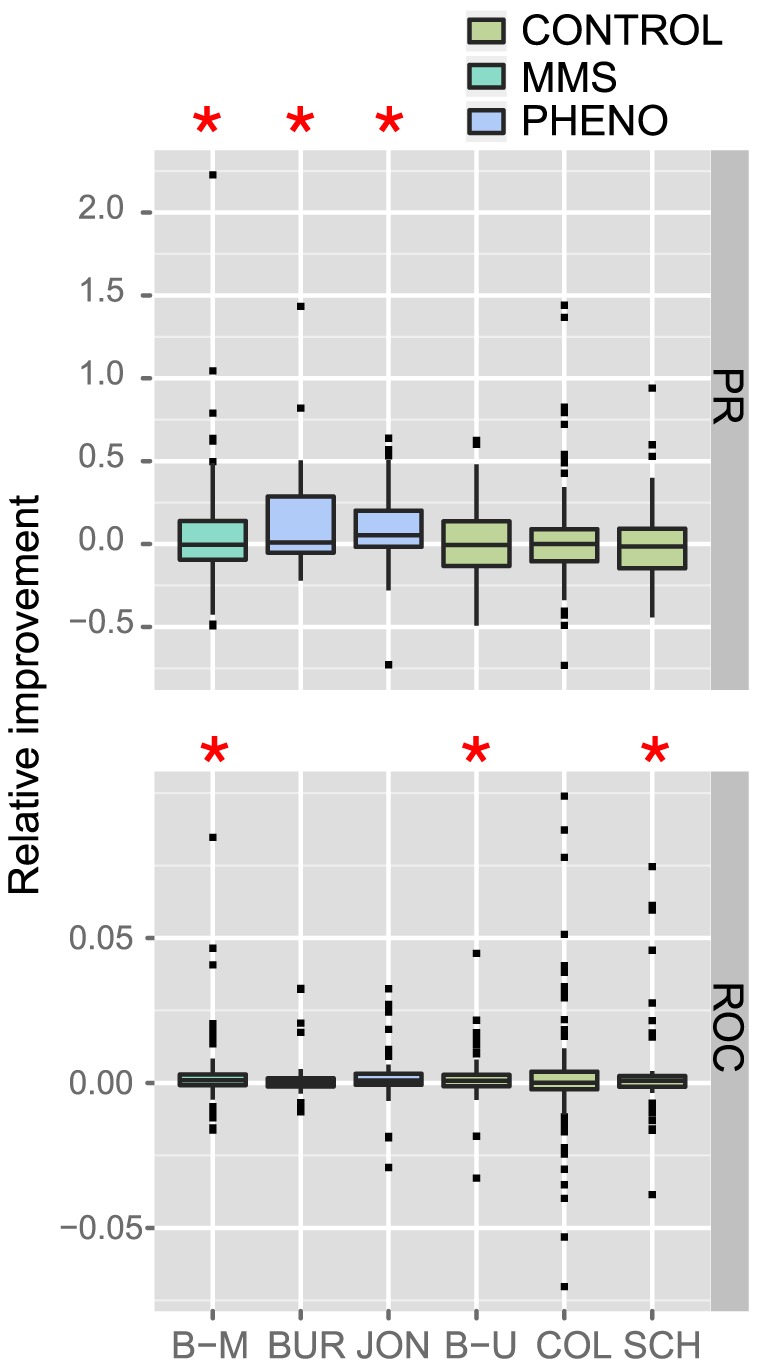
Complementarity of the networks as measured by gene function prediction. The boxplots show the relative improvement of the area under the receiver operating characteristic (ROC) and the precision recall (PR) curves obtained when predicting gene function with the GeneMANIA algorithm on the Gene Ontology categories when combining each network with the reference, in comparison to predicting with each network separately. The red stars indicate a significant improvement (p-value<0.05). The networks are B–M Bandyopadhyay et al. [9] in MMS, B–U Bandyopadhyay et al. [9] untreated, BUR Burston et al. [5], JON Jonikas et al. [14], SCH Schuldiner et al. [16], COL Collins et al. [15].

We find that PHENO/MMS networks each enable a significant performance improvement in PR values when combined with the reference network (Figure 3A, Table 1), whereas CONTROL networks do not provide a significant improvement. The difference between PHENO/MMS and CONTROL is highly significant (Wilcoxon p-value<0.0043). This suggests that the unique information provided by the PHENO/MMS networks is complementary to the information from the reference network and combining them improves gene function prediction.

**Table 1:** Relative area under the precision-recall (PR) curve improvement for all considered GO terms.

		PHENO/MMS			CONTROL		
PR improvement	GLOBAL	BMS	BUR	JON	BUN	COL	SHU
# terms	496	81	49	47	81	179	59
# positive	250	37	27	33	38	88	27
# negative	243	44	22	14	43	88	32
mean	0.044	0.071	0.118	0.093	0.014	0.028	−0.004
p-value	**2.2E-4**	**0.04**	**0.004**	**0.0037**	0.27	0.078	0.55

Significant p-values (<0.05) are bolded.

However the ROC results are less clear (Figure 3B) where the set of networks providing significantly complementary information (Schuldiner, Bandyopadhyay-mms and Bandyopadhyay-un) does not correspond directly to the set of PHENO/MMS networks. Also, when considering all networks, gene function prediction performance is improved when combining a given network with the reference both for PR (Table 1, p<2.2e-4) and ROC (Table 2, p<7.6e-5). This suggests that other factors, such as laboratory effects, may also contribute to the presence of complementary information.

**Table 2:** Relative area under the receiver-operating characteristic (ROC) curve improvement for all considered GO terms.

		PHENO/MMS			CONTROL		
ROC improvement	GLOBAL	BMS	BUR	JON	BUN	COL	SHU
# terms	496	81	49	47	81	179	59
# positive	300	55	28	33	54	94	36
# negative	192	25	21	14	27	82	23
mean	0.0024	0.0031	0.0016	0.0028	0.0019	0.0021	0.0039
p-value	**7.6E-05**	**0.016**	0.08	0.053	**0.022**	0.057	**0.047**

Significant p-values (<0.05) are bolded.

To investigate the differences between the combined networks and the reference, we selected the GO terms with the highest gene function prediction PR value differences (adjusted p-value<0.05) (Figure S2). We found that Burston performs significantly better on ‘actin filament organization’, ‘late endosome to vacuole transport via multivesicular body sorting pathway’ and ‘endoplasmic reticulum unfolded protein response’ (Figure S3). The members of the ‘actin filament organization’ biological process are more densely connected in the correlation network in the Burston data set leading to better gene function prediction as compared to the reference SGA data set where PBS2 is not connected at all. The Jonikas data set performs better on ‘protein glycosylation’ and ‘Hrd1p ubiquitin ligase ERAD-L complex’ (Figure S4). For the latter complex, the subunits are generally better connected in the Jonikas dataset, leading to better gene function prediction for this GO term. For instance, Jonikas shows a strong correlation between YOS9 and HRD3 subunits, which physically interact, but this correlation is not strong in the reference. Similarly, the members of the lipid-linked oligosaccharide biosynthesis pathway (ALG9, ALG6, ALG3, ALG12) are strongly connected in the Jonikas data set, leading to better gene function prediction for this GO term. Jonikas shows strong correlations between those four genes, which all physically interact, but those correlations are not present in the SGA reference. For the control networks, Collins performs better on ‘loop DNA binding’, ‘mismatch repair’ and ‘histone exchange’ while Schuldiner is worse on ‘dolichyl-diphosphooligosaccharide-protein glycotransferase activity’ and ‘Hrd1p ubiquitin ligase ERAD-L complex’. Both Bandyopadhyay networks (untreated and in presence of MMS) perform better on ‘regulation of transcription’ but the untreated network performs worse on ‘regulation of cyclin-dependent protein kinase activity’ (it only contains one correlation between MIH1 and PTC3 protein phosphatase genes, while the reference contains many more correlations (Figure S5). ROC values did not distinguish GO terms enough to identify significant differences between networks (Figure S6).

As noted above, it is possible that function-based gene selection in PHENO, MMS and CONTROL networks could bias our results. In particular, gene selection bias causes a different set of GO terms to be tested for each network. Thus, we repeated our gene function prediction analysis on triplets of gene pairs tested across SGA, PHENO/MMS and CONTROL networks. The combination of the PHENO/MMS correlation network with the reference correlation network tends to perform better in terms of gene function prediction as compared to that of the CONTROL and reference networks (Figure S7), for example for ‘response to stress’ in both PR and ROC measurements (Text S1). As before the trend is significant on the PR measurements (paired Wilcoxon test p<0.012) but not on the ROC measurements.

Altogether, our results show that genetic interactions mapped in different conditions provide complementary information.

### Comparison across all networks reveals an effect associated to the laboratory

**Figure 4:**
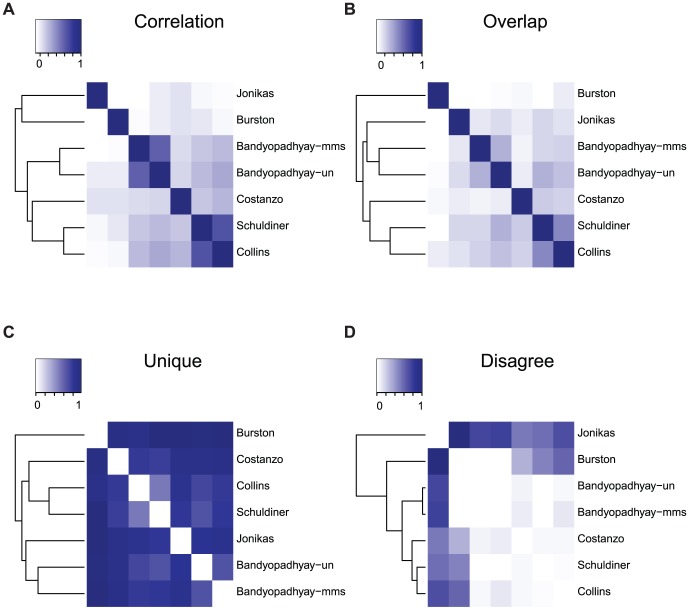
Comparison of all networks. The comparison measures (A: Correlation, B: Overlap, C: Unique, D: Disagree) between all pairs of networks considered in the study are shown in a clustered heat map view.

The above results hinted that there may exist factors other than phenotypic readout or condition that explain genetic interaction data set differences. To gain a better understanding of these potential other factors, we generalized our analysis to compare all pairs of networks, by clustering the all data set by all data set comparison matrices for our four measures: correlation, overlap, unique and disagree. The two networks obtained with different phenotypes (Burston and Jonikas) are clearly outliers in this analysis, in particular for the correlation values (Figure 4A), reinforcing our above results. Surprisingly, the Bandyopadhyay et al. MMS network is always grouped with its associated untreated network, which are both separated from the control networks and very close to each other (4A–D). Indeed their correlation (r = 0.58) is the second highest in the correlation matrix. This suggests that factors, such as the laboratory environment external to the experiment, also affect network mapping. This may be due to the ‘batch effect’ recently described for large-scale genetic interactions [22]. In agreement with this, the most correlated networks (Schuldiner and Collins, r = 0.65) were obtained in the same laboratory. Since these two networks are both in the control group (similar phenotype, similar conditions), we were originally not surprised to find that they are always grouped together. However, the fact that they are more similar each other than they are to the SGA network suggests an important laboratory effect is present. As an additional analysis, we compared genetic interaction profiles for individual genes across all data sets (Methods). For a given gene and a given pair of networks, we computed the correlation (Spearman) between the genetic interaction profiles of that gene in both networks. This measure was previously used, for example, to identify genes with different profiles between untreated and DNA damage condition genetic interaction networks [9]. Clustering all networks based on their average correlation measures across all genes shows similar results to those above (Figure S8). Thus, in addition to phenotypic readout and internal experiment condition, external factors in the laboratory where the experiment is performed contribute to the unique information present in each network.

### Combining all networks improves gene function prediction

**Figure 5:**
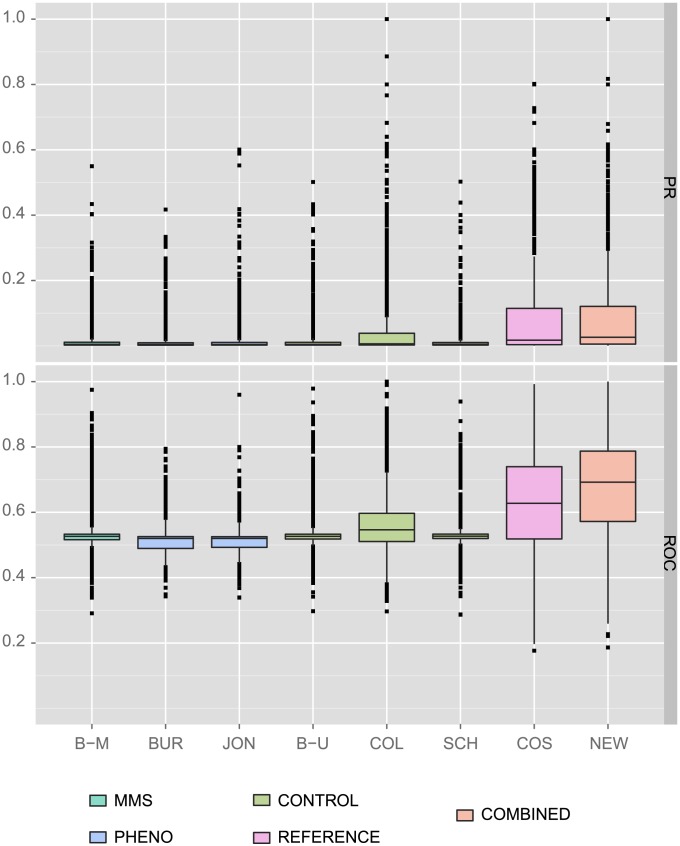
Gene function prediction results on full independent and combined networks. The boxplots show the area under the receiver operating characteristic (ROC) curves obtained when predicting gene function with the GeneMANIA algorithm on the Gene Ontology categories for the networks separately and after combination, using all available genes and interactions (full networks): B–M Bandyopadhyay et al. [9] in MMS, B–U Bandyopadhyay et al. [9] untreated, BUR Burston et al. [5], JON Jonikas et al. [14], SCH Schuldiner et al. [16], COL Collins et al. [15], COS Costanzo et al. [3], NEW the combined network.

To create a fair comparison, we previously reduced each set of networks analyzed to common tested gene pairs. However, all of the information available in all networks should be considered for gene function prediction. Thus, we repeated our analysis of gene function prediction performance using genetic interaction profile correlation networks computed using all genes in each data set and combined all seven of them using the same correlation network building methodology described above (max correlation). We find that the combined network provides substantially better results, on average, across GO terms for both ROC and PR performance measures (Figure 5).

**Figure 6:**
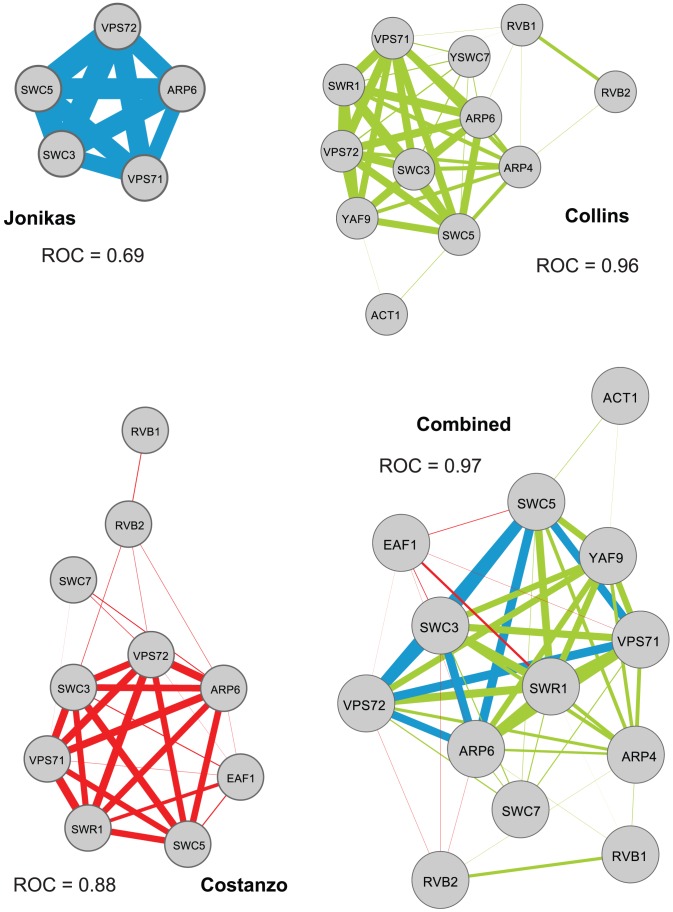
The SWR1 complex is better predicted when combining multiple networks. Nodes represent genes and edges represent genetic interaction profile correlations between the genes that are part of the SWR1 complex (GO:0000812). All of its 13 subunits are connected when combining all networks, whereas only subsets of those are connected in each individual network. Networks were visualized using Cytoscape [33].

To illustrate the complementarity of the individual correlation networks, we examined the SWR1 complex, one of the annotation categories that the combined network predicts better than any individual network (Figure 6). The SWR1 complex (GO:0000812) is a multi-subunit complex involved in chromatin remodeling and is required for the incorporation of the histone variant H2AZ into chromatin. All of its 13 subunits are connected when combining all networks, whereas only subsets of those are connected in each individual network (five genes in Jonikas et al., 10 in Costanzo et al., 12 in Collins et al.). In some cases the missing genes were not present in the original screen (Jonikas and Costanzo), while in others they were mostly present (Collins), illustrating the benefit of the new combined network to gather information and genes from different studies to get a more complete view of functional connections among all genes in a system.

## Discussion

Genetic interaction experiments are performed using a particular phenotypic readout and set of experimental conditions in a given species. Using recently available data, we conducted a systematic analysis of quantitative genetic interaction networks in budding yeast mapped under different experimental conditions. We showed that genetic interaction networks mapped in different environmental and laboratory conditions or using different phenotypic readouts provide unique and complementary information. The functional interactions defined by genetic interaction profile correlations can be combined using a simple ‘max correlation’ procedure to aid gene function prediction.

Given the low overlap between the data sets, we adopted a reference-based comparison approach where each data set is in turn compared to a common high confidence reference. While this enables a global comparison, it is possible that the reference network is biased towards certain gene sets present in only some compared networks and this could affect our results. Thus, we repeated our analysis on a set of gene pairs present across three networks under comparison. While these results agree, there a many fewer gene pairs tested across three networks than there are for two networks. The SGA dataset continues to grow and will be complete in the future. Also, we expect additional networks to be mapped under different conditions. Ideally, an additional global genetic interaction map of the scale of SGA in different conditions would be available to analyze, but this is unlikely to be available anytime soon, as SGA cost millions of dollars and has already taken more than a decade to achieve a 30% coverage rate of all interactions. Smaller genetic interaction networks mapped under different environment and phenotypic readout among comparable gene sets are more likely to be available in the near future and would help test our results.

We propose a simple method to combine diverse genetic interaction networks and show that this improves gene function prediction. We chose to combine data sets at the level of genetic interaction profile correlations instead of individual genetic interactions for a number of reasons: correlation can be computed for all gene pairs in a sufficiently large genetic interaction map not just those pairs tested in both maps, no tuning of parameters is needed, no normalization of individual data sets is needed as would be required if combining data at the level of genetic interactions [7], correlation is the primary type of relationship used for gene function prediction from genetic interaction networks [3], [18], and similar methods are established in the gene expression field that we can draw from [23]. We chose gene function prediction as a means to assess and compare the biological content of each network, as it is one of the main goals of genetic interaction mapping. However, other measures could be used such as the overlap with benchmark data sets [7]. Moreover, it is likely that the method we propose could be improved to yield even better gene function prediction results, for instance by tuning the weight of each network to optimize gene function prediction for a given gene function, as is done in the multi-network version of GeneMANIA [19] (we only used GeneMANIA on a single combined genetic interaction profile correlation network). It will also be interesting to evaluate the gene function prediction improvement gained by combining genetic interactions with other types of network data, such as protein-protein interactions. We provide our combined network as a resource at http://baderlab.org/Data/GeneticInteractionComparison.

We expect our results to extend to other organisms, which are increasingly targeted for genetic interaction mapping [24]–[30] with traditional growth assays and diverse phenotypic readouts [31]. Analysis of additional multi-condition and multi-phenotype data will eventually enable us to select experimental conditions that maximize discovery of gene function information, as has been accomplished with gene expression data [32].

## Methods

### Genetic interaction networks

All genetic interaction data sets were downloaded from original publications or requested from the authors (Figure 1, Text S1).

### Measures to compare a network to the reference

The measures used to compare a network to the reference are: ‘correlation’ is the Spearman correlation coefficient of genetic interaction scores for all compared pairs; ‘overlap’ is the percentage of binary interactions in common among all observed interactions; ‘unique’ is the percentage of interactions observed in only one network among all observed interactions; ‘disagree’ is the percentage of interactions of different type (positive, negative) among all interactions observed in common. Gene profile correlation is computed for a given gene as the Spearman correlation coefficient of the genetic interaction profiles of that gene in two data sets, limited to genetic interaction partners found in both data sets. The similarity between two data sets used for clustering is the mean of the gene profile correlation distribution (Figure S3). We only consider gene pairs tested in all data sets to enable a fair comparison. For the stochastic model, we use the error rates estimated by Costanzo et al. for positive (sensitivity = 0.18 and precision = 0.59) and negative (sensitivity = 0.35 and precision = 0.63) genetic interactions. Since such estimates for the other data sets are not available, we use the Costanzo values for all data sets. This information is then used to compute the expected number of interactions present in zero, one or two data sets and compared to the observed numbers of interactions (Text S1). We compare those measures between networks in the CONDITION group to networks in the CONTROL group with a Student's t-Test.

### Gene function prediction assessment

To limit the analysis to the best associations, correlation networks only contain correlation values higher than 0.1. To assess each network, we use the command line version of the GeneMANIA Cytoscape plugin (version 2.11) [20]. We use five-fold cross validation with the function ‘CrossValidator’ and then compared the results for the different networks. The validation was run on a set of 3618 GO terms (1789 BP, 1299 MF, 530 CC), though only a subset of these terms are tested in each network (according to which genes are present). To avoid circularity in the analysis and annotations potentially coming from the networks we are studying, we only considered annotations that were derived from direct assays/experiments (evidence codes EXP, IDA, IPI, IMP, IGI, IEP). We manually checked that IGI annotations were not derived from genetic interactions from networks we analyze (only three IGI annotations from these studies were found). For both the PR and ROC assessments, each network is associated with a score. The relative improvement of the combined network C obtained from two individual networks A and B is computed as follows:
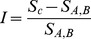
where 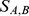 is the mean score of the two individual networks A and B.

## Supporting Information

Figure S1 Similarity measures restricted to the sets of gene pairs tested in the reference, a CONTROL and a PHENO/MMS network. For a given measure, the difference between the PHENO/MMS and CONTROL values is tested by a paired t-test. For the specific case with Bandyopadhyay-MMS as PHENO/MMS and Schuldiner as CONTROL (BMS-SHU), no interactions are observed between the same gene pairs, thus the agreement coefficient is not available.
(EPS)

Figure S2 Performance of the combined and reference networks as measured by the area under the PR curve.
(EPS)

Figure S3 Correlation networks for the SGA and Burston data sets, limited to the gene pairs tested in both. The color of the edges indicates the network. The thicker the edge, the higher the correlation value.
(EPS)

Figure S4 Correlation networks for the SGA and Jonikas data sets, limited to the gene pairs tested in both. The color of the edges indicates the network. The thicker the edge, the higher the correlation value.
(EPS)

Figure S5 Correlation networks for the SGA and Bandyopadhyay networks, limited to the gene pairs tested in both. The color of the edges indicates the network. The thicker the edge, the higher the correlation value.
(EPS)

Figure S6 Performance of the combined and reference networks as measured by the area under the ROC curve.
(EPS)

Figure S7 Improvement in the gene function prediction when combining either the PHENO/MMS or the CONTROL correlation network with the SGA reference correlation network, on the exact same set of gene pairs for all three networks.
(EPS)

Figure S8 Clustering of the data sets based on the gene profile correlation values. The hierarchical clustering was done using different criteria (Ward, Complete, Average, Median).
(EPS)

Text S1 This document contains more detailed information about the genetic interaction networks, the comparison measures and the gene function prediction performance.
(PDF)
