# Supplementary material for: Multiple Genetic Interaction Experiments Provide Complementary Information Useful for Gene Function Prediction
Source: PLoS Comput Biol. 2012 Jun 21;8(6):e1002559. doi: 10.1371/journal.pcbi.1002559 (PMC3380825; doi:10.1371/journal.pcbi.1002559)
Supplement: Text S1 — This document contains more detailed information about the genetic interaction networks, the comparison measures and the gene function prediction performance. (PDF) [file pcbi.1002559.s009.pdf]

Supplementary material

Multiple genetic interaction experiments provide complementary information useful for gene function prediction

Magali Michaut and Gary D. Bader

GENETIC INTERACTION NETWORKS **2**
DATA SETS 2
DEFINITION OF THE COMMON SPACE 2
FILTERING INTERACTIONS 2
COMPARISON MEASURES DEFINITION **2**
OVERLAP AND AGREEMENT BETWEEN DATA SETS 2
STATISTICAL MODEL 3
COMPARISON MEASURES RESULTS **6**
ALL TESTED GENE PAIRS 6
TRIPLETS OF GENE PAIRS TESTED ACROSS REFERENCE, CONTROL AND CONDITION 6
GENE FUNCTION PREDICTION PERFORMANCE **8**
ALL TESTED GENE PAIRS 8
TRIPLETS OF GENE PAIRS TESTED ACROSS REFERENCE, CONTROL AND CONDITION 13
SOFTWARE **17**
REFERENCES **17**

Genetic interaction networks

Data sets

All genetic interaction data sets were downloaded from original publications or requested from the authors. When comparing two data sets, we only consider gene pairs tested in both. All networks were considered as undirected (query and array genes where reported have the same role in our analysis).

Network	Name	Genes	Positive interactions	Negative interactions	Positive cutoff	Negative cutoff
Costanzo [1]	COS	4417	56138	101256	0.08	-0.08
Collins [2]	COL	743	5690	11482	1.9	-2.5
Schuldiner [3]	SCH	424	1278	3679	2.0	-2.0
Bandyopadhyay-un [4]	B-U	419	957	2027	1.5	-2.0
Bandyopadhyay-mms [4]	B-M	419	864	2069	1.8	-2.2
Burston [5]	BUR	359	711	1103	5463	-4681
Jonikas [6]	JON	330	1196	1911	0.81	-0.38

Table S1. Description of the genetic interaction data sets

Definition of the common space

In order to perform a meaningful comparison between given data sets, we consider only gene pairs that were tested in all of them. We filter out genes that were not present in both studies.

Filtering interactions

The SGA data set is defined as the intermediate data set in Costanzo et al. (epsilon>0.08 and p<0.05). We define the cutoffs for other data sets so that the numbers of positive and negative observed interactions are the same as for SGA.

	COS	COL	SCH	B-U	B-M	BUR	JON
COS	5276435	101908	37255	40331	40331	10100	17038
COL	101908	183125	398	4916	4916	548	1558
SCH	37255	398	89676	43	43	781	5429
B-U	40331	4916	43	78841	78841	212	341
B-M	40331	4916	43	78841	78841	212	341
BUR	10100	548	781	212	212	20795	334
JON	17038	1558	5429	341	341	334	34634

Table S2. Number of pairs tested in both data sets.

Comparison measures definition

Overlap and agreement between data sets

When comparing data sets, we only consider gene pairs tested in all sets. For each gene pair, we consider if it was observed as interacting in zero, one or two data sets. When it is observed in both data sets, we then consider the type of interaction (positive/negative) and check if both data sets agree on this type. 'Overlap' is the percentage of interactions in common among all observed interactions; 'unique' is the percentage of interactions

observed in only one network among all observed interactions; 'disagree' is the percentage of interactions of different type (positive, negative) among all interactions observed in common.

P-value Estimate	COS	COL	SCH	B-U	B-M	BUR	JON
COS		0	0	1.40E-125	2.80E-72	1.60E-09	3.40E-09
COL	2.20E-01		2.90E-98	1.20E-193	1.20E-82	1.30E-01	6.40E-05
SCH	1.40E-01	6.50E-01		8.00E-02	1.60E-01	4.60E-01	1.10E-06
B-U	8.40E-02	2.90E-01	1.90E-01		0	8.20E-01	8.90E-01
B-M	6.30E-02	1.90E-01	1.50E-01	5.80E-01		1.20E-01	2.80E-02
BUR	4.20E-02	-4.50E-02	1.90E-02	-1.10E-02	-7.50E-02		7.30E-03
JON	3.20E-02	-7.20E-02	-4.70E-02	-5.20E-03	-8.40E-02	-1.00E-01	

Table S3. Correlation matrix (lower part is Spearman estimate and upper part is p-value).

COS							
COL	0.218						
SCH	0.229	0.487					
B-U	0.122	0.274	0.333				
B-M	0.091	0.224	0.2	0.327			
BUR	0.074	0.127	0.05	0.069	0.031		
JON	0.127	0.166	0.189	0.182	0.145	0.034	
	COS	COL	SCH	B-U	B-M	BUR	JON

Table S4. Overlap matrix.

COS							
COL	0.78						
SCH	0.77	0.51					
B-U	0.88	0.73	0.67				
B-M	0.91	0.78	0.8	0.67			
BUR	0.93	0.87	0.95	0.93	0.97		
JON	0.87	0.83	0.81	0.82	0.85	0.97	
	COS	COL	SCH	B-U	B-M	BUR	JON

Table S5. Unique matrix.

COS							
COL	0.05						
SCH	0.06	0.02					
B-U	0.06	0.05	1				
B-M	0.08	0.11	1	0.01			
BUR	0.31	0.62	0.50	1	1		
JON	0.53	0.72	0.57	0.75	0.78	0	
	COS	COL	SCH	B-U	B-M	BUR	JON

Table S6. Disagreement matrix.

Statistical model

Definitions and Notations

	Interacting |I|=n	Non Interacting |Ic|=m
Edge (=observed)	TP	FP

No edge (=not observed)	FN	TN

Table S7. Definitions and notations.

True positive (TP) are interacting gene pairs connected by an edge.
False positive (FP) are not interacting gene pairs connected by an edge.
True negative (TN) are not interacting gene pairs not connected by an edge.
False negative (FN) are interacting gene pairs not connected by an edge.
Sensitivity = TP / (TP + FN) = True positive rate (TPR) = Recall
Precision = TP / (TP + FP) = True discovery rate (TDR)
Probability of FP (PFP) = FP rate (FPR) = FP / (FP + TN)
Probability of FN (PFN) = FN rate (FNR) = FN / (FN + TP)

Error rate estimation

Based on symmetrical interactions and known functional relationships, Costanzo et al. assessed the error rates in the SGA data set for positive and negative interactions (Table S8).

Network	Number interactions	Sensitivity	Precision
$\varepsilon>0.08$, $p<0.05$	59,887	0.18	0.59
$\varepsilon<-0.08$, $p<0.05$	108,417	0.35	0.63

Table S8. Sensitivity and precision of SGA genetic interactions scores [1].

The probability to have FN (or false negative rate) is directly given by the sensitivity
$PFN = 1 - sensitivity$

Nevertheless, the probability to have FP is not directly given by the precision. Given the number of tested and observed interactions in the common space and using these values of sensitivity and precision, we can compute the estimated numbers of TP, TN, FP, FN and finally compute the probabilities to have FP as follows:

The number of gene pairs with an edge is $observed = TP + FP$

Thus using the definition of the precision, the number of TP is:
$TP = precision * (TP + FP) = precision * observed$

We can deduce FP:
$FP = observed - TP$

Using the definition of the sensitivity, we compute the number of FN as:
$FN = TP * (1 - sensitivity) / sensitivity$

And finally we get TN:
$TN = tested - (TP + FP + FN)$
$PFP = FP / (FP + TN)$

Unfortunately, we don't know the error rates for other data sets. Consequently, we used the values from the SGA data set as an estimate of these rates. We define the cutoff so that the numbers of observed interactions match between the two data sets (if both data sets are sampled from the same model and with the same error rates, we expect the same numbers of interactions on the common space).

Expected counts

Using the notations of Chiang et al. [7], we define gene pairs as being interacting (I) or not interacting (Ic). Given the values n=|I| and m=|Ic|, we can define the expected values of three random variables: the number of gene pairs where no edge exists in any data set (X0), the number of gene pairs where an edge exists for exactly one data sets (and not the other)(X1), the number of gene pairs where an edge exists in both data sets (X2).

$$E[X_0] = n * P_{FN}^{A} * P_{FN}^{B} + m * (1 - P_{FP}^{A})(1 - P_{FP}^{B}) \quad (1)$$

$$\begin{aligned} E[X_1] &= n * ((1 - P_{FN}^{A}) * P_{FN}^{B} + (1 - P_{FN}^{B}) * P_{FN}^{A}) \\ &+ m * (P_{FP}^{A} * (1 - P_{FP}^{B}) + P_{FP}^{B} * (1 - P_{FP}^{A})) \end{aligned} \quad (2)$$

$$E[x_2] = n * (1 - P_{FN}^{A})(1 - P_{FN}^{B}) + m * P_{FP}^{A} * P_{FP}^{B} \quad (3)$$

The total number of gene pairs considered is N = n+m.

Comparison of observed and expected

We consider here the presence or absence of an interaction between each tested gene pair. The overlap is measured by the Jaccard coefficient (intersection / union).

Network			Overlap				Unique			
Group	Name	Type	Exp	Obs	D	Pval	Exp	Obs	D	Pval
CONT	COL	pos	0.059	0.098	+	2.2e-11	0.94	0.90	-	5.6e-05
CONT	COL	neg	0.130	0.260	+	1.1e-69	0.87	0.74	-	6.9e-70
CONT	SCH	pos	0.058	0.077	+	4.0e-02	0.94	0.92	-	1.7e-01
CONT	SCH	neg	0.128	0.278	+	1.2e-26	0.87	0.72	-	2.0e-27
CONT	B-U	pos	0.058	0.060	+	4.6e-01	0.94	0.94	-	4.6e-01
CONT	B-U	neg	0.126	0.143	+	1.3e-01	0.87	0.86	-	1.8e-01
MMS	B-M	pos	0.058	0.039	-	4.9e-02	0.94	0.96	+	2.3e-01
MMS	B-M	neg	0.126	0.106	-	6.0e-02	0.87	0.89	+	1.2e-01
PHENO	BUR	pos	0.059	0.035	-	3.9e-02	0.94	0.96	+	2.0e-01
PHENO	BUR	neg	0.130	0.058	-	8.7e-07	0.87	0.94	+	9.5e-04
PHENO	JON	pos	0.060	0.032	-	1.4e-03	0.94	0.97	+	9.5e-02
PHENO	JON	neg	0.130	0.070	-	3.0e-07	0.87	0.93	+	3.7e-04

Table S9. Comparison of expected and observed measures between the reference SGA network and other networks separated by type (positive/negative interactions). The column D indicates if the observed overlap/unique measure is more (+) or less (-) than

expected. P-values are computed using a Fisher's exact test between expected and observed counts.

Comparison measures results

All tested gene pairs

For two given network groups, we test the difference of the means of each given measure with a Student's t-Test. When there is a single network in the group (MMS) we assess the significance using a normal distribution with mean and standard deviation estimated from the control distribution, which is assumed to be normally distributed (no rejection of the Shapiro test).

group1	group2	measure	p-value
PHENO / MMS	CONTROL	correlation	0.055810537
PHENO / MMS	CONTROL	overlap	0.047527726
PHENO / MMS	CONTROL	unique	0.047527726
PHENO / MMS	CONTROL	disagree	0.095747104
PHENO / MMS	CONTROL	pos overlap	0.022831834
PHENO / MMS	CONTROL	pos unique	0.022400195
PHENO / MMS	CONTROL	neg overlap	0.027792196
PHENO / MMS	CONTROL	neg unique	0.027815372
PHENO	CONTROL	correlation	0.050406387
PHENO	CONTROL	overlap	0.064920128
PHENO	CONTROL	unique	0.064920128
PHENO	CONTROL	disagree	0.092633679
PHENO	CONTROL	pos overlap	0.021599608
PHENO	CONTROL	pos unique	0.020868108
PHENO	CONTROL	neg overlap	0.027834032
PHENO	CONTROL	neg unique	0.027880324
MMS	CONTROL	correlation	0.103693128
MMS	CONTROL	overlap	0.046250538
MMS	CONTROL	unique	0.046250538
MMS	CONTROL	disagree	5.44E-13
MMS	CONTROL	pos overlap	0.015150213
MMS	CONTROL	pos unique	0.016404722
MMS	CONTROL	neg overlap	0.047406139
MMS	CONTROL	neg unique	0.048687717

Table S10. Indicative statistics on the comparison of different groups of networks regarding the comparison measures computed (correlation, overlap, unique, disagree).

Triplets of gene pairs tested across reference, control and condition

We consider here only gene pairs that were tested in the reference network and in a PHENO/MMS network and a CONTROL network. There are a total of 48499 of these triplets of gene pairs.

REF	PHENO/MMS	CONTROL	Shared Genes	Shared Tested Interactions
SGA	Bandyopadhyay-mms	Collins	115	3137
SGA	Bandyopadhyay-mms	Schuldiner	9	23
SGA	Bandyopadhyay-mms	Bandyopadhyay-un	382	40331
SGA	Burston	Collins	65	379
SGA	Burston	Schuldiner	57	471
SGA	Burston	Bandyopadhyay-un	35	107
SGA	Jonikas	Collins	69	929
SGA	Jonikas	Schuldiner	104	2945
SGA	Jonikas	Bandyopadhyay-un	33	177

Table S12. Number of gene pairs that were tested in the SGA reference network, one of the PHENO/MMS networks and one of the CONTROL networks.

We computed the similarity measures on the subset of gene pairs present in a given triplet of networks described in Table S12.

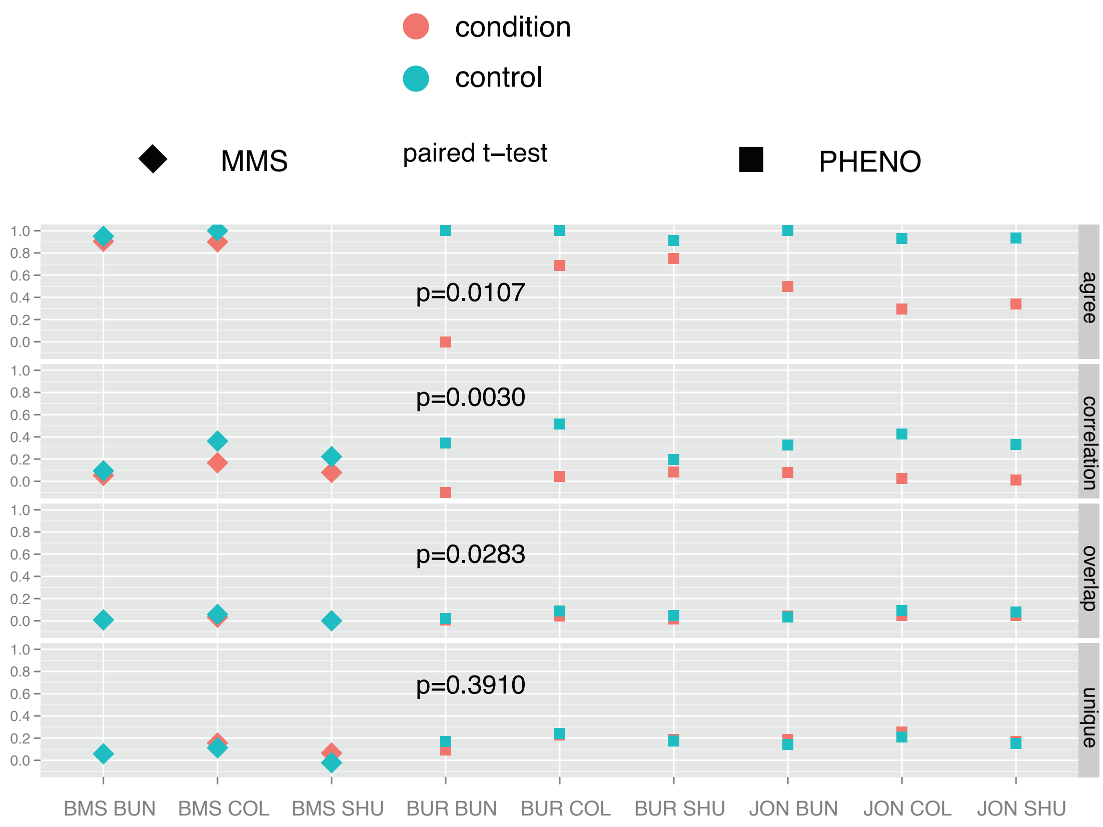

Figure S1. Similarity measures restricted to the sets of gene pairs tested in the reference, a CONTROL and a PHENO/MMS network. For a given measure, the difference between the PHENO/MMS and CONTROL values is tested by a paired t-test. For the specific

case with Bandyopadhyay-MMS as PHENO/MMS and Schuldiner as CONTROL (BMS-SHU), no interactions are observed between the same gene pairs, thus the agreement coefficient is not available.

Gene function prediction performance

For each network, we only consider GO terms with at least five genes in the networks.

All tested gene pairs

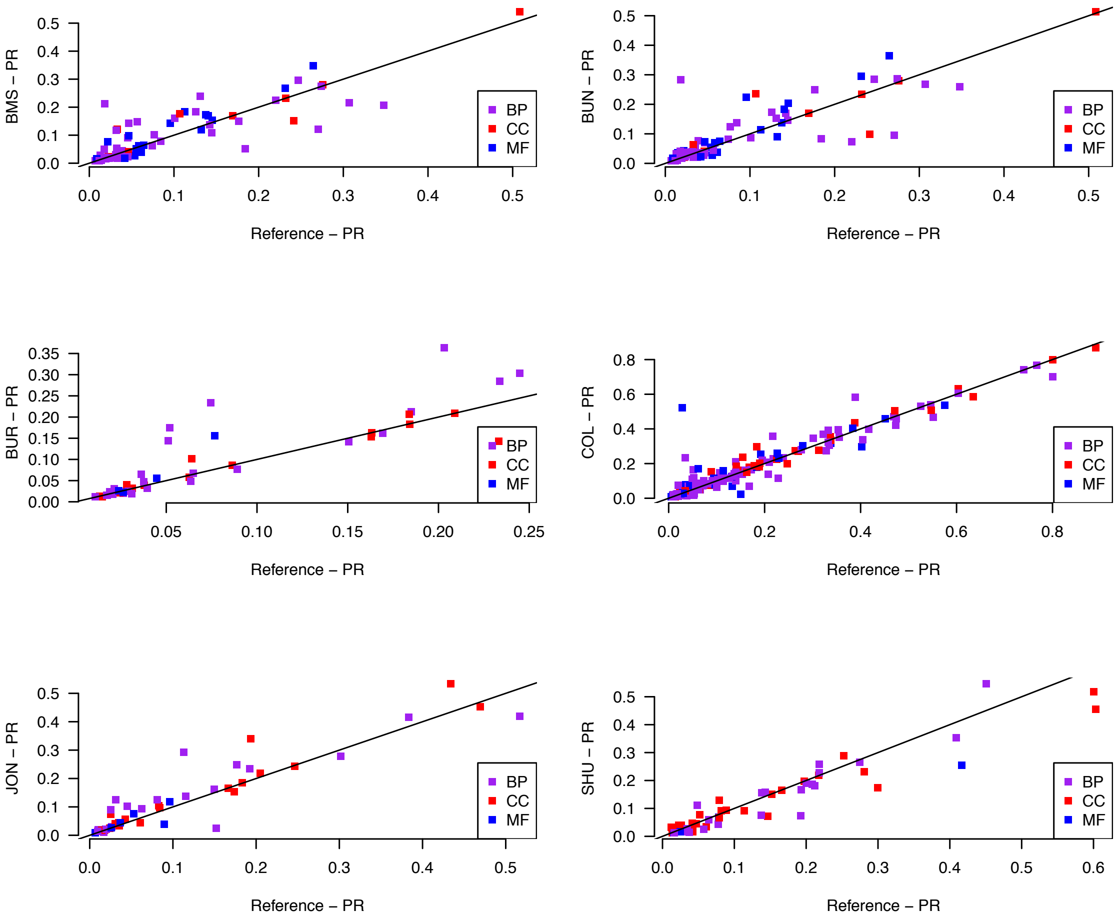

Figure S2. Performance of the combined and reference networks as measured by the area under the PR curve.

GO identifier	GO Term Name	Network	Size	Improvement score	Adjusted p-value
GO:0045449	regulation of transcription	BMS	7	0.19	0.004

GO:0000079	regulation of cyclin-dependent protein kinase activity	BUN	6	-0.18	0.026
GO:0045449	regulation of transcription	BUN	7	0.26	4.93E-05
GO:0007015	actin filament organization	BUR	9	0.12	0.039
GO:0030968	endoplasmic reticulum unfolded protein response	BUR	5	0.16	0.003
GO:0032511	late endosome to vacuole transport via multivesicular body sorting pathway	BUR	7	0.16	0.003
GO:0000404	loop DNA binding	COL	5	0.49	3.81E-17
GO:0006298	mismatch repair	COL	10	0.20	0.012
GO:0043486	histone exchange	COL	10	0.19	0.012
GO:0000839	Hrd1p ubiquitin ligase ERAD-L complex	JON	5	0.15	0.039
GO:0006486	protein glycosylation	JON	9	0.18	0.007
GO:0000839	Hrd1p ubiquitin ligase ERAD-L complex	SHU	5	-0.15	0.015
GO:0004579	dolichyl-diphosphooligosaccharide-protein glycotransferase activity	SHU	6	-0.16	0.010

Table S11. Improvement of the gene function performance of the combined network as compared to the condition and reference networks alone as measured by the area under the PR curve. The relative improvement of the combined network C obtained from two individual networks A and B is computed as follows:

$$I = \frac{S_c - S_{A,B}}{S_{A,B}}$$

where $S_{A,B}$ is the mean score of the two individual networks A and B. Significant outliers were identified based on their residuals to the linear fit. P-values were then computed under the assumption that the distribution of the residuals is normal, and were further corrected for multiple testing using the Benjamini-Hochberg method (FDR < 0.05).

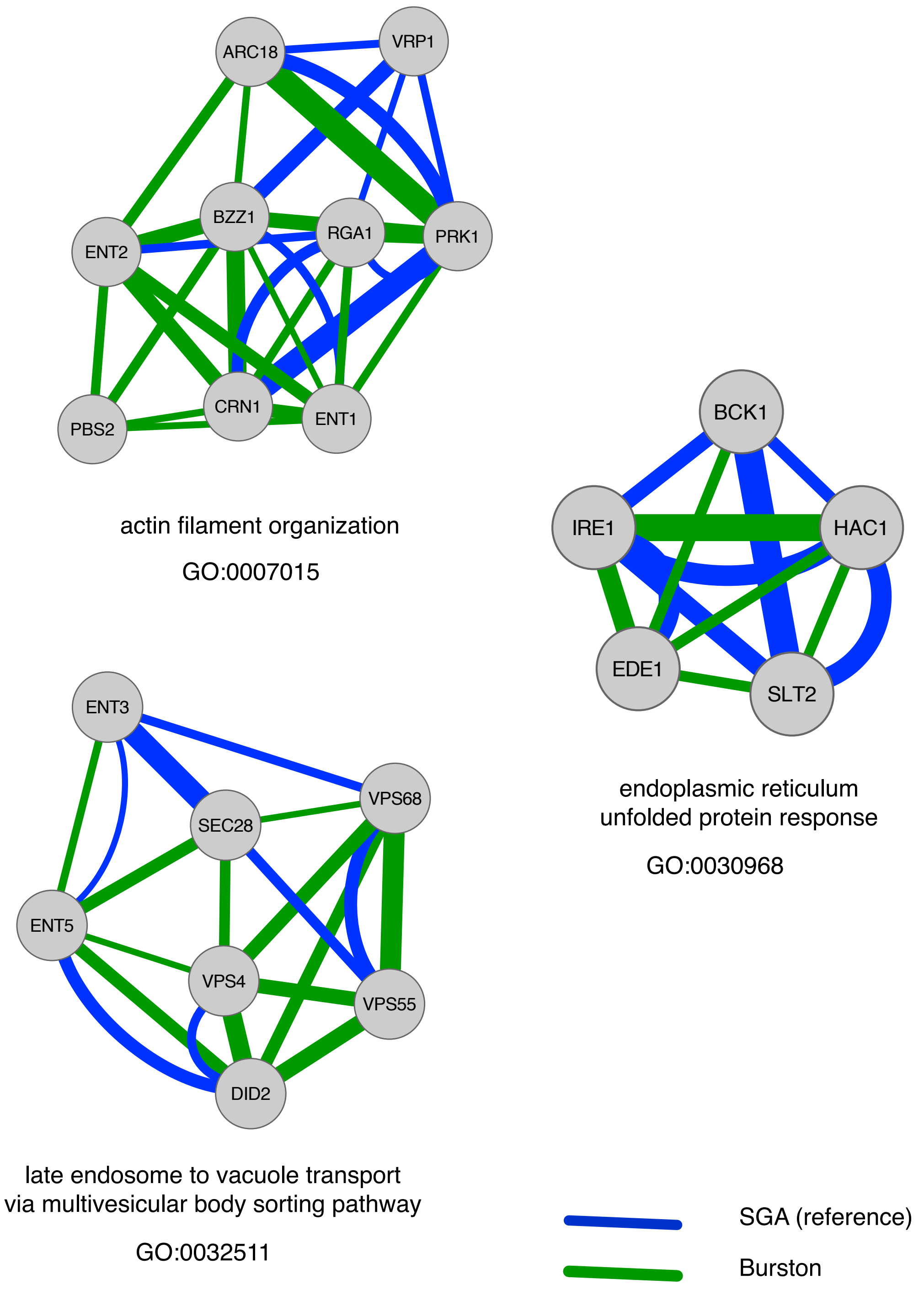

Figure S3. Correlation networks for the SGA and Burston data sets, limited to the gene pairs tested in both. The color of the edges indicates the network. The thicker the edge, the higher the correlation value.

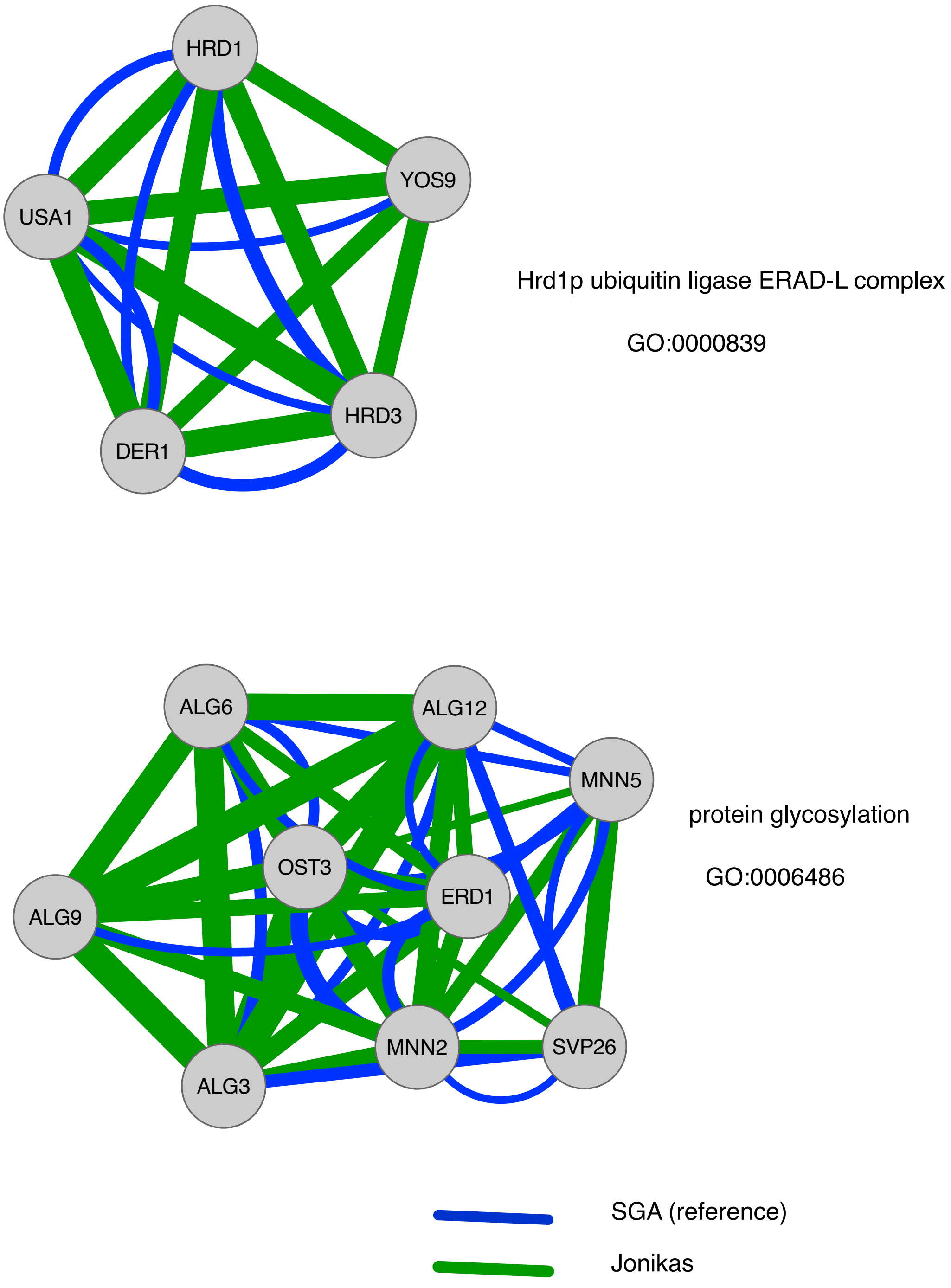

Figure S4. Correlation networks for the SGA and Jonikas data sets, limited to the gene pairs tested in both. The color of the edges indicates the network. The thicker the edge, the higher the correlation value.

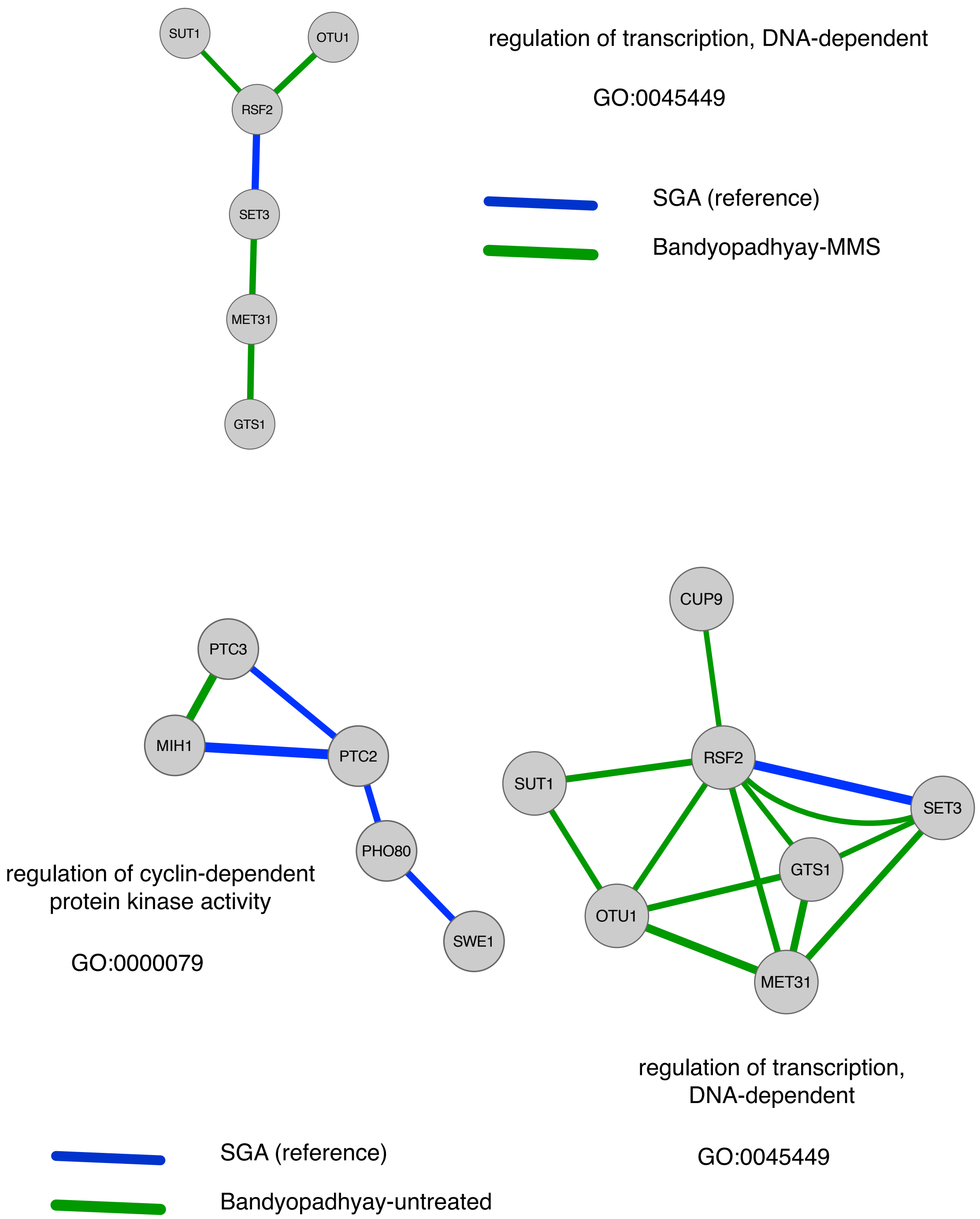

Figure S5. Correlation networks for the SGA and Bandyopadhyay networks, limited to the gene pairs tested in both. The color of the edges indicates the network. The thicker the edge, the higher the correlation value.

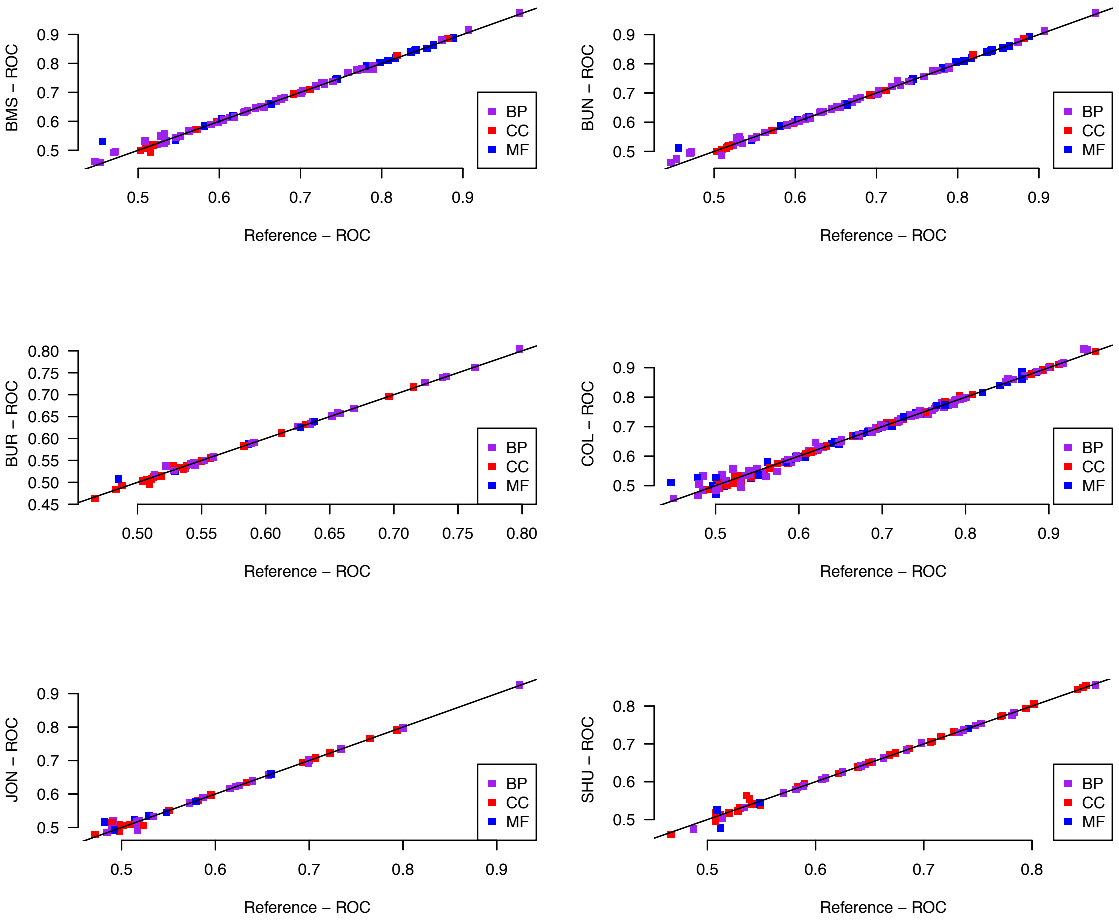

Figure S6. Performance of the combined and reference networks as measured by the area under the ROC curve.

Triplets of gene pairs tested across reference, control and condition

We study gene function prediction performance on the sets of interactions present in three data sets as described above (triplets). We consider the gene function performance when combining the PHENO/MMS to the SGA reference and when combining the CONTROL to the reference in order to assess the complementarity of the networks. The relative improvement of the combined network C obtained from two individual networks A and B is computed as:

$$I = \frac{S_c - S_{A,B}}{S_{A,B}}$$

where $S_{A,B}$ is the mean score of the two individual networks A and B.

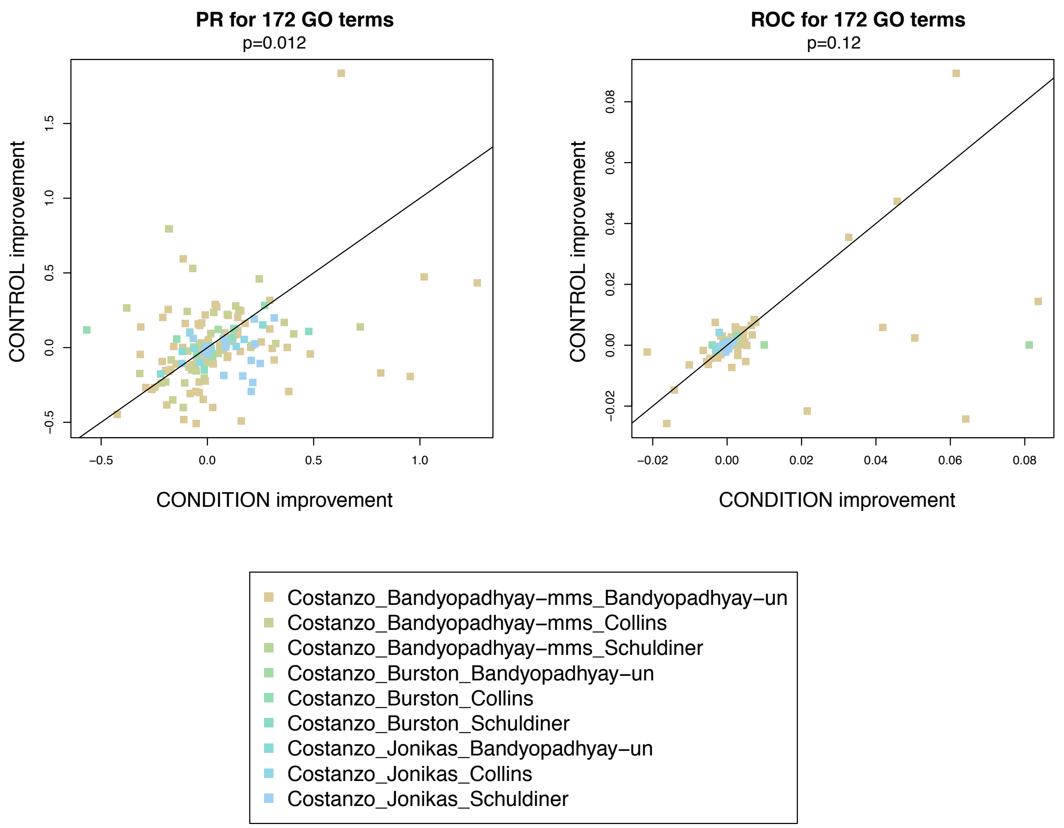

Figure S7. Improvement in the gene function prediction when combining either the PHENO/MMS or the CONTROL correlation network with the SGA reference correlation network, on the exact same set of gene pairs for all three networks.

GO		CONDITION		CONTROL		Diff
Id	Name	Network	Improvement	Network	Improvement	
GO:0006950	response to stress	BMS	0.0641	BUN	-0.0242	0.088
GO:0004674	protein serine/threonine kinase activity	Burston	0.0812	BUN	9.61E-05	0.081
GO:0003682	chromatin binding	BMS	0.0836	BUN	0.0144	0.069
GO:0006348	chromatin silencing at telomere	BMS	0.0503	BUN	0.0024	0.047
GO:0030437	ascospore formation	BMS	0.0215	BUN	-0.0215	0.043
GO:0034599	cellular response to oxidative stress	BMS	0.0418	BUN	0.0057	0.036
GO:0005886	plasma membrane	BMS	0.0050	BUN	-0.0052	0.010
GO:0006468	protein phosphorylation	Burston	0.0099	BUN	0.0001	0.009

Table S13. List of GO terms with the highest difference in gene function prediction improvement in the CONDITION versus the CONTROL data sets for all interactions tested in the triplet networks (in ROC measurements).

GO		CONDITION		CONTROL		Diff
Id	Name	Network	Improvement	Network	Improvement	
GO:0007165	signal transduction	BMS	0.9548	BUN	-0.1948	1.149
GO:0010553	negative regulation of gene-specific transcription from RNA polymerase II promoter	BMS	0.8152	BUN	-0.1699	0.985
GO:0006950	response to stress	BMS	1.2700	BUN	0.4340	0.836
GO:0006631	fatty acid metabolic process	BMS	0.3837	BUN	-0.2947	0.678
GO:0000082	G1/S transition of mitotic cell cycle	BMS	0.1584	BUN	-0.4913	0.649
GO:0004407	histone deacetylase activity	BMS	0.7187	Collins	0.1365	0.582
GO:0006350	transcription	BMS	1.0194	BUN	0.4732	0.546
GO:0003702	RNA polymerase II transcription factor activity	BMS	0.4851	BUN	-0.0420	0.527
GO:0005788	endoplasmic reticulum lumen	Jonikas	0.2060	Schuldiner	-0.2937	0.499

Table S14. List of GO terms with the highest difference in gene function prediction improvement in the CONDITION versus the CONTROL data sets for all interactions tested in the triplet networks (in PR measurements).

Gene profile correlations

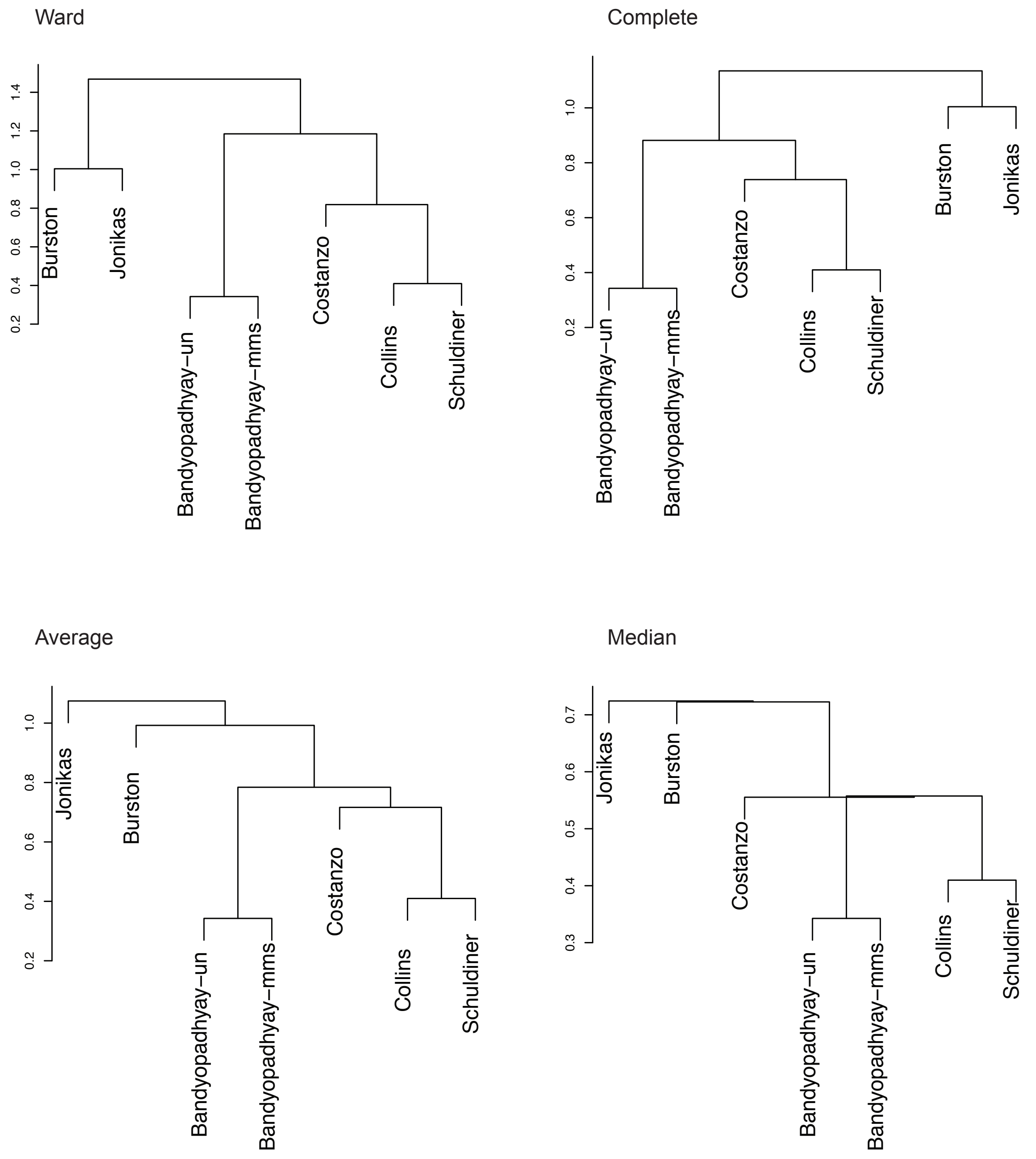

Figure S8. Clustering of the data sets based on the gene profile correlation values. The hierarchical clustering was done using different criteria (Ward, Complete, Average, Median).

Software

The analyses were performed with R/Bioconductor [8,9], the libraries gplots [10] and ggpplots2 [11], as well as the annotation packages GO.db [12] and org.Sc.sgd.db [13].

References

1. Costanzo M, Baryshnikova A, Bellay J, Kim Y, Spear ED, et al. (2010) The genetic landscape of a cell. Science 327: 425-431.
2. Collins SR, Miller KM, Maas NL, Roguev A, Fillingham J, et al. (2007) Functional dissection of protein complexes involved in yeast chromosome biology using a genetic interaction map. Nature 446: 806-810.
3. Schuldiner M, Collins SR, Thompson NJ, Denic V, Bhamidipati A, et al. (2005) Exploration of the function and organization of the yeast early secretory pathway through an epistatic miniarray profile. Cell 123: 507-519.
4. Bandyopadhyay S, Mehta M, Kuo D, Sung M-K, Chuang R, et al. (2010) Rewiring of genetic networks in response to DNA damage. Science 330: 1385-1389.
5. Burston HE, Maldonado-Báez L, Davey M, Montpetit B, Schluter C, et al. (2009) Regulators of yeast endocytosis identified by systematic quantitative analysis. J Cell Biol 185: 1097-1110.
6. Jonikas MC, Collins SR, Denic V, Oh E, Quan EM, et al. (2009) Comprehensive characterization of genes required for protein folding in the endoplasmic reticulum. Science 323: 1693-1697.
7. Chiang T, Scholtens D, Sarkar D, Gentleman R, Huber W (2007) Coverage and error models of protein-protein interaction data by directed graph analysis. Genome Biol 8: R186.
8. R Development Core Team (2011). R: A language and environment for statistical computing. R Foundation for Statistical Computing, Vienna, Austria. ISBN 3-900051-07-0, URL http://www.R-project.org/.
9. Gentleman RC, Carey VJ, Bates DM, Bolstad B, Dettling M, et al. (2004) Bioconductor: open software development for computational biology and bioinformatics. Genome biology 5: R80.
10. Gregory R. Warnes. Includes R source code and/or documentation contributed by: Ben Bolker (in alphabetical order), Lodewijk Bonebakker, Robert Gentleman, Wolfgang Huber Andy Liaw, Thomas Lumley, Martin Maechler, Arni Magnusson, Steffen Moeller, Marc Schwartz and Bill Venables (2011). gplots: Various R programming tools for plotting data. R package version 2.10.1. http://CRAN.R-project.org/package=gplots.
11. H. Wickham. ggplot2: elegant graphics for data analysis. Springer New York, 2009.
12. Marc Carlson, Seth Falcon, Herve Pages and Nianhua Li (). GO.db: A set of annotation maps describing the entire Gene Ontology. R package version 2.5.0.
13. Marc Carlson, Seth Falcon, Herve Pages and Nianhua Li (). org.Sc.sgd.db: Genome wide annotation for Yeast. R package version 2.5.0.
